# *OsMADS1* Represses microRNA172 in Elongation of Palea/Lemma Development in Rice

**DOI:** 10.3389/fpls.2016.01891

**Published:** 2016-12-20

**Authors:** Zhengyan Dai, Jiang Wang, Mulan Zhu, Xuexia Miao, Zhenying Shi

**Affiliations:** Key Laboratory of Insect Developmental and Evolutionary Biology, Institute of Plant Physiology and Ecology, Shanghai Institutes for Biological Sciences, Chinese Academy of SciencesShanghai, China

**Keywords:** *OsMADS1*, miR172, floral organs, lemma, palea, determinacy

## Abstract

Specification of floral organ identity is critical for the establishment of floral morphology and inflorescence architecture. Although multiple genes are involved in the regulation of floral organogenesis, our understanding of the underlying regulating network is still fragmentary. MADs-box genes are principle members in the ABCDE model that characterized floral organs. *OsMADS1* specifies the determinacy of spikelet meristem and lemma/palea identity in rice. However, the pathway through which *OsMADS1* regulates floral organs remains elusive; here, we identified the microRNA172 (miR172) family as possible regulators downstream of *OsMADS1*. Genetic study revealed that overexpression of each miR172 gene resulted in elongated lemma/palea and indeterminacy of the floret, which resemble the phenotype of *osmads1* mutant. On the contrary, overexpression of each target *APETALA2* (*AP2*) genes resulted in shortened palea/lemma. Expression level and specificity of miR172 was greatly influenced by *OsMADS1*, as revealed by Northern blot analysis and *In situ* hybridization. Genetically, *AP2-3* and *AP2-2* over expression rescued the elongation and inconsistent development of the lemma/palea in OsMADS1RNAi transgenic plants. Our results suggested that in rice, *OsMADS1* and miR172s/*AP2*s formed a regulatory network involved in floral organ development, particularly the elongation of the lemma and the palea.

## Introduction

Specification of floral meristem fate and floral organ identity is pivotal for the reproductive development of plants, molecular, and genetic studies led to the establishment of the classic ABC and the modified ABCDE model to explain the development of floral organs (Coen and Meyerowitz, 1991; Angenent et al., 1995; Pelaz et al., 2000; Ditta et al., 2004). MADs-box genes are characterized by the presence of an approximately 60 amino acids DNA-binding MADS-box domain in the N-terminal (Schwarz-Sommer et al., 1990; Theissen et al., 2000; Arora et al., 2007). A considerable number of MADs-box genes exist in plants which are major players in the control of flower architecture, flower induction, and vegetative development, the phylogeny of MADs-box genes is tightly correlated with the evolution of plant reproductive structures (Theissen et al., 2000; Ferrario et al., 2004). Quite a lot of A/B/C/D/E class genes have been identified, and all of them except for *AP2* genes are MADs-box family genes. Rice belongs to the grass family of monocots, the florets of which contain carpels and stamens, but lack petals and sepals, instead, lodicules surround the sex organs and the lemma/palea envelop the inner floral organs. Similarly, MADs-box genes, such as *OsMADS1, OsMADS3* and *OsMADS58* (Yamaguchi et al., 2006), *OsMADS6* (Ohmori et al., 2009; Li et al., 2010), *OsMADS7* and *OsMADS8* (Cui et al., 2010), and *OsMADS15* (Wang K. et al., 2010) characterize floral organ identities in rice.

Among them, the function of *OsMADS1* is extensively studied due to identification of several mutants (Jeon et al., 2000; Agrawal et al., 2005; Chen et al., 2006; Wang K. et al., 2010). The *naked seed rice* (*nsr*) mutant displayed overdeveloped lemma and palea, the transformation of lodicules into palea/lemma-like organs, and decreased number of stamens (Chen et al., 2006). The *leafy hull sterile1* (*lsh1*) mutant produces spikelets consisting of elongated leafy paleae and lemmas, two pairs of leafy palea-like and lemma-like lodicules, a decrease in stamen number and an increase in the number of carpels, some spikelets generate an additional floret from the same rachilla (Jeon et al., 2000). The *afo* mutant is an epigenetic mutation in *OsMADS1* that showed pleiotropic defects in lemmas and the inner three whorls and the distinct “flower-in-flower” structure (Wang K. et al., 2010). Altogether, *OsMADS1* mutations result in over developed lemma/palea, transition of the inner three whorls into lemma/palea structures and loss of flower determinacy, suggesting a role for *OsMADS1* in specifying the determinacy of the flower meristem and influencing development of all floral organs. In accordance, sequence and function conservation allotted *OsMADS1* gene to the E-function gene (Agrawal et al., 2005; Cui et al., 2010).

*OsMADS34* and *OsMADS55* are two downstream genes of *OsMADS1*; with *OsMADS34* characterizing the spikelet meristem and *OsMADS55* function in organ differentiation (Khanday et al., 2013). *OsMGH3* might be an indirect downstream gene of *OsMADS1* (Prasad et al., 2005). However, *OsMADS1* is a regulator of genetic networks that orchestrate transcriptional and signaling pathways to promote rice floret specification and development, the molecular mechanism downstream of *OsMADAS1* is still not so clear, and complex physical and genetic interaction might exist between *OsMADS1* and other floral organ characterizing genes, most of which are also MADS-box genes (Hu et al., 2015; Khanday et al., 2016).

In recent years, microRNAs (miRNAs) have been shown to play pivotal regulative roles in many developmental and physiological processes in a wide variety of organisms (Chapman and Carrington, 2007; Molnar et al., 2007; Zhao T. et al., 2007; Xie and Qi, 2008; Zhou et al., 2013). In plants, miR172 is involved in the regulation of flowering time and floral organ identity through targeting *AP2* genes (Chen, 2004; Mlotshwa et al., 2006; Zhao L. et al., 2007; Martin et al., 2009; Mathieu et al., 2009). *AP2* genes encode plant specific transcriptional factors which are characterized by the AP2 DNA-binding domain (Yaish et al., 2010). *AP2* genes form a big super-family in plants, and play various roles in plant development and physiology (Chen, 2004; Nakano et al., 2006), such as in floral organ development (Chen, 2004), in response to biotic and abiotic stress (Tang et al., 2005; Shukla et al., 2006), and in seed size control (Fu and Xue, 2010). In rice, one target of miR172, *supernumerary bract* (*SNB*), influences the floral organ identity and floral determinacy (Lee et al., 2007). Another target of miR172, *Osindeterminate spikelet 1* (*OsIDS1*) functions in the establishment of the floral meristems and formation of the floral organs (Lee and An, 2012). miR172 expresses in the late vegetative stage and panicle, and over expression of miR172b could approximately pheno-copy the *snb* mutant (Zhu et al., 2009). Although miR172 is functionally conserved, and several studies strongly suggest the participation of miR172/*AP2*s in floral organ development in rice, a comprehensive understanding of their roles is still missing.

In the present study, the functions of all four miR172s and the five target *AP2*s in rice were analyzed. Over expression of the respective miR172s caused elongation of the lemma/palea and loss of floral determinacy, resembling the phenotype of the *osmads1* mutant. Consistently, overexpression of each target *AP2* gene resulted shortened lemma/palea to various degrees, suggesting that miR172/*AP2*s regulate the elongation of the lemma/palea. In *OsMADS1* RNAi transgenic plants, the four miR172s were up-regulated, whereas in *OsMADS1* overexpression transgenic plants, the four miR172s were down-regulated. Furthermore, the timing and specificity of miR172 expression were both influenced by *OsMADS1*. Genetically, overexpression of *AP2-3* and *AP2-2* partially rescued the phenotype of OsMADS1RNAi. These results strongly suggested that miR172/*AP2*s regulated palea/lemma development and floral determinacy in rice, and *OsMADS1* was an upstream suppressor of miR172.

## Materials and Methods

### Plant Materials

*Oryza sativa japonica* variety Zhonghua No.11 (abbreviated as ZH11) was used as wild-type. All the plants used in this study were grown in the green house with an 8 h light and 16 h dark cycle; or in a paddy field under natural conditions in summer.

### Construction of Transgenic Plasmids and Genetic Transformation

For overexpression of miR172s, genomic fragments containing the Osa_miR172s coding regions were, respectively, cloned into the pCAMBIA1301 under the 35S promoter and NOS terminator (p130135SNOS).

For overexpression of *AP2s* and *OsMADS1*, the full length cDNA of the respective genes were cloned into the p130135SNOS vector.

For overexpression of *AP2-2* in the OsMADS1RNAi plants, the whole expression cassette of 35s-cDNA-nos was digested from the p130135SNOS-AP2-2 and cloned in the p230135SNOS vector, using *Hind*III and *Eco*RI.

For *OsMADS1* RNAi construction, a gene-specific cDNA fragment was cloned into the p1301RNAi vector in the sense orientation using *Bam*HI and *Kpn*I and antisense orientation using *Sac*I and *Spe*I.

Plasmid p2301-AP2-2 was transformed into OsMADS1RNAi plants with G418 selection, other plasmids were transformed into ZH11 using *Agrobacterium*-mediated genetic transformation with hygromycin selection, respectively (Hiei et al., 1994).

### Reverse Transcription-Polymerase Chain Reaction (RT-PCR) and Real Time RT-PCR Analysis

Total RNAs were extracted from the leaves or young panicles of the plants using the TRIzol (Invitrogen), and then reverse transcribed using ReverAce (TOYOBO). cDNA was synthesized from 2 μg of total RNA treated with DNase I (TOYOBO) and used as template.

### *In situ* Hybridization and miRNA *In situ* Hybridization

Young panicles were fixed in 4% paraformaldehyde PBS solution (0.1% Triton-X-100, 0.1% Tween-20, 4% formaldehyde, 25% glutaraldehyde) overnight at 4°C, dehydrated through a concentration grade of ethanol, cleared through a dimethylbenzene series, infiltrated through a series of paraffin (Sigma-Aldrich), and finally embedded in 100% paraffin melted at 60°C. The samples were sectioned longitudinally at 7 μm and then mounted on RNase-free glass slides (Sigma). A gene-specific region of *OsMADS1* was cloned into the pBSK(-) vector, linearized, and used as template for amplifying digoxigenin-labeled sense and antisense RNA probes using a DIG RNA labeling kit (Promega). *In situ* hybridization was performed as previously described (Coen et al., 1990).

For miRNA *in situ* hybridization, materials were prepared as usual. miR172 was detected with Locked Nucleic Acid (LNA) probes which were Digoxin 5′-end labeled (Exiqon).

### miRNA Northern Blot Hybridization

Approximately 30 μg of total RNA was separated on 15% polyacrylamide denaturing gels. RNAs were transferred to Amersham Hybond^®^-N^+^ membranes and cross-linked by UV irradiation; the membranes were hybridized with biotin-labeled DNA probes complementary to the miRNA sequences at 42°C overnight. The membranes were then washed and incubated with a stabilized streptavidin-horseradish peroxidase at 42°C. After washing with substrate equilibration buffer and adding stable peroxide solution and enhancer solution, the membranes were imaged using an FLA-5000 Phosphorimager. The DNA probes were synthesized and biotin-labeled using a 3′ end DNA labeling method.

### Scanning Electron Microscope (SEM) Analysis

Shoot apical meristems and IMs and young florets were decorticated under light microscope and fixed quickly in 50% FAA at 4°C overnight after vacuuming, and dehydrated through a graded concentration of ethanol. For SEM analysis, the samples were then critical point dried using liquid carbon dioxide and mounted on SEM stubs, sputter coated with gold and palladium (4:1) and examined using a SEM (Hitachi S-2460, Japan). For paraffin analysis, samples were embedded in epoxide resin and cut into slices 2–3 μm slices; strips of these slices were spread at 42°C on a hot platform overnight, stained using 0.5% toluidine Blue O and sealed for observation under the microscope (Wang J.et al., 2010).

### Yeast One-Hybrid Assay

The full length cDNA of *OsMADS1* gene was cloned in frame into vector pPC86.

PCR fragments containing the binding motifs of *OsMADS1* were, respectively, cloned into the p178 vector using the *Xho*I restriction site, which contains the *CYC1* core promoter and the *lacZ* gene.

Yeast strain EGY48 (*MATtrp1his3ura3leu2*::*6lexAop*-*LEU2*) was used for transformation. The yeast assays were performed according to the manufacturer’s protocol with the substrate chlorophenol red-D-galactopyranoside (Matchmaker One-hybrid System; Clontech).

### Yeast Two-Hybrid Assay

The open reading frame (ORF) of *OsMADS1* was amplified and cloned into the prey vector pGAD-T7. The ORFs of AP2s were amplified and cloned into the bait vector pGBK-T7. The yeast two-hybrid assay was performed according to the manufacturer’s instructions (Clontech).

### Sequence Information

Sequence data used in this study can be found in the rice genome annotation database^1^ and NCBI^2^ under the following accession numbers: LOC_Os03g11614 (*OsMADS1*), LOC_Os05g03040 (*AP2-1*), LOC_Os03g60430 (*AP2-2*), LOC_Os07g13170 (*AP2-3*), LOC_Os06g43220 (*AP2-4*), LOC_Os04g55560 (*AP2-5*), LM379345 (Osa-miR172a), LM379346 (Osa-miR172b); LM379347 (Osa-miR172c), LM383079 (Osa-miR172d), AC091532 (*actin*).

Primer sequences used in this study are listed in Supplementary Table S1.

## Results

### Overexpression of Each miR172 Gene Caused Elongated Lemma/Palea and Indeterminacy

**FIGURE 1:**
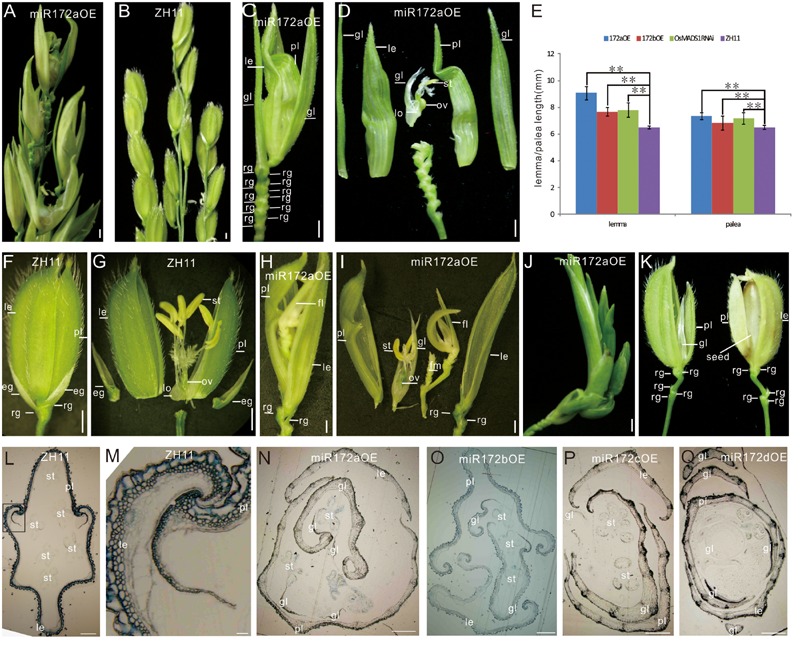
**Phenotypes of miR172OEs plants. (A,B)** Half panicles of the miR172aOE and ZH11 plants, respectively. **(C,F)** A spikelet of the miR172aOE plant and ZH11 plant, respectively. **(D,G)** Dissection of the spikelet in **(C,F)**, respectively. **(E)** Statistical analysis of the length of the lemma and palea in the transgenic plants. Double asterisks represent significant difference determined by the Student’s *t*-test at ^∗∗^*P* < 0.01. **(H,I)** Full view and dissection of the spikelet of miR172aOE plants. **(J)** A plantlet-like spikelet of miR172aOE plants. **(K)** A spikelet (left) and a grain (right) of miR172bOE plants. **(L)** Transverse section of ZH11 floret. **(M)** Zoom in of the palea/lemma interlocking region in **(L)** (rectangle). **(N–Q)** Transverse section of the florets of miR172a, miR172b, miR172c, and miR172dOE plants. Bars in **(A–D,F–J)** were 1 mm, bars in **(L–Q)** were 0.5 cm. Abbreviations: le, lemma; gl, glume; pl, palea; rg, rudimentary glume; eg, empty glume; lo, lodicule; ov, ovule; st, stamen; sti, stigma; fl, floret; fm, floral meristem.

Four miR172 genes (miR172a-d) have been identified in rice genome^3^. To study their function, we, respectively, over expressed them under the 35S promoter through genetic transformation. For each miR172 over expression, 30–50 independent transgenic plants were gotten and over 90% of them showed the phenotypes described below. The expression levels of respective miR172s in the transgenic lines were verified by Northern blotting (Supplementary Figure S1). According to the severity of the phenotype, the transgenic plants from different miR172s overexpression could be grouped into two classes: miR172aOE, miR712cOE, and miR172dOE were similar and showed severely abnormal floral organs (**Figure 1A** as compared with **Figure 1B**), they were tentatively represented as miR172aOEs unless specified; whereas phenotypic abnormality of miR172bOE was moderate (see the following).

The miR172aOEs plants showed elongated lemma and palea, with the lemma longer than the palea (**Figures 1C–E**). Typically, a wild-type rice floret consists of a lemma and a palea in whorl1, and two lodicules at the lemma side in whorl2, six stamens in whorl3, and a carpel with a pair of feather-like stigmas and a green ovary in whorl4. A floret together with two pairs of sterile glumes (empty glumes and rudimentary glumes) constitutes a spikelet (**Figure 1F**). In the greenhouse, the lemma/palea of wild-type averaged 6.5 mm, whereas the lemma and palea of the miR172aOE was 9.07 and 7.34 mm, respectively, the lemma and palea of miR172bOE averaged 7.68 and 6.82 mm, respectively (**Figure 1E**).

miR172aOEs plants produced many rudimentary glumes (**Figure 1C**) instead of one pair in the wild-type (**Figure 1F**); and showed “flower-in-flower” structures (**Figures 1H,I**); the extreme phenotype was an overall plantlet-like structure instead of a flower (**Figure 1J**). All these characters indicated that miR172aOEs plants showed indeterminacy in flower development.

Phenotype of miR172bOE plants was more moderate than that of miR172aOEs. The lemma of miR172bOE was less elongated (**Figures 1E,K**); fewer ectopic glumes and rudimentary glumes were formed; and some flowers were fertile (**Figure 1K**).

Normally, the lemma and palea of wild-type plants were closed (**Figure 1F**) except for the short opening time during flowering. However, in the four miR172OE plants, the lemma and palea could not close due to severe distortion (**Figures 1N–Q** as compared with **Figure 1L**). As a result, the normal interlocking structure between the lemma and palea in the wild-type (**Figure 1M**) disappeared in the four miR172OE plants (**Figures 1N–Q**).

**FIGURE 2:**
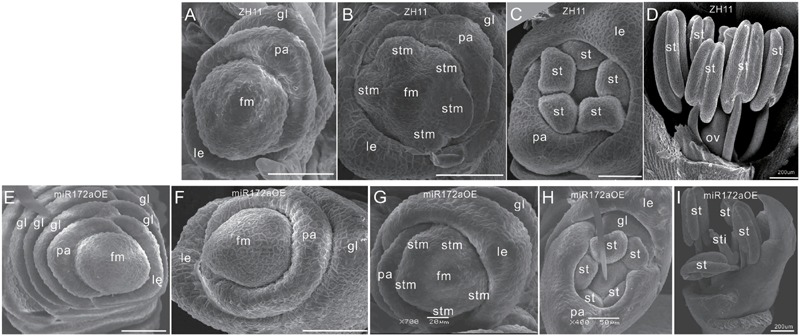
**Floret development in miR172aOEs plants. (A–D)** and **(F–I)** Spikelet meristem, stamen meristem, floret meristem, and floret of ZH11 and miR172aOE plants, respectively. **(E)** Disturbed determination of the miR172aOE spikelet meristem. Bars were 50 μm unless specified. Abbreviations: SAM, shoot apical meristem; P0, leaf meristem; gl, glume; pa, palea; le, lemma; fm, floral meristem; stm, stamen meristem; st, stamen; ov, ovule; sti, stigma; ca, carpel.

Furthermore, SEM analysis revealed that the transition from spikelet meristem to floral meristem was delayed in the miR172aOEs plants. In wild-type floret, after differentiation of a pair of rudimentary glumes and empty glumes, the floral meristem begins to development inner floral organs (**Figures 2A–D**). In the miR172aOEs plants, the meristem produced many rudimentary glumes before forming a floret (**Figures 1C and 2E**). In the process of floral meristem development, before the formation of the stamen, no visible phenotypic changes were observed (**Figure 2F** compared with **Figure 2A**, **Figure 2G** compared with **Figure 2B**), but after that, the palea/lemma elongated differently (**Figure 2H**); and ectopic glumes inside the palea/lemma (**Figure 2H** as compared with **Figure 2C**) and malformed stamens (**Figure 2I**) developed. In most of the florets, stamens were decreased and malformed (**Figures 1D,I**), miR172aOEs plants were infertile.

We further cloned AtmiR172a precursor from *Arabidopsis* and overexpressed it in rice. Mature AtmiR172a was the same as the OsmiR172a although their precursor differs (Supplementary Figure S2). The AtmiR172aOE lines showed elongated and distorted palea/lemma (indicated by stars) and indeterminacy of flowers, as indicated by increased number of rudimentary glumes (arrows), similar to the phenotype of OsmiR172aOEs (Supplementary Figure S3). This further illustrated the conservation of miR172.

### Overexpression of Each Target *AP2* Gene Resulted in Shortened Lemma/Palea

To study the function of miR172 in detail, we further analyzed the function of miR172 targets genetically. In rice, five *AP2* genes are predicted as targets of miR172^4^ (Zhu et al., 2009), and they were tentatively designated as *AP2-1, AP2-2, AP2-3, AP2-4*, and *AP2-5*, respectively, in this study. All the five *AP2* genes have two AP2 DNA-binding domains in a similar mode (Supplementary Figure S4). To mimic the knock-down function of miR172, these *AP2* genes were, respectively, overexpressed. 30–50 individual transgenic plants were gotten for each gene, and over 90% of them showed the respective phenotype illustrated below, which proved the successful genetic transformation.

**FIGURE 3:**
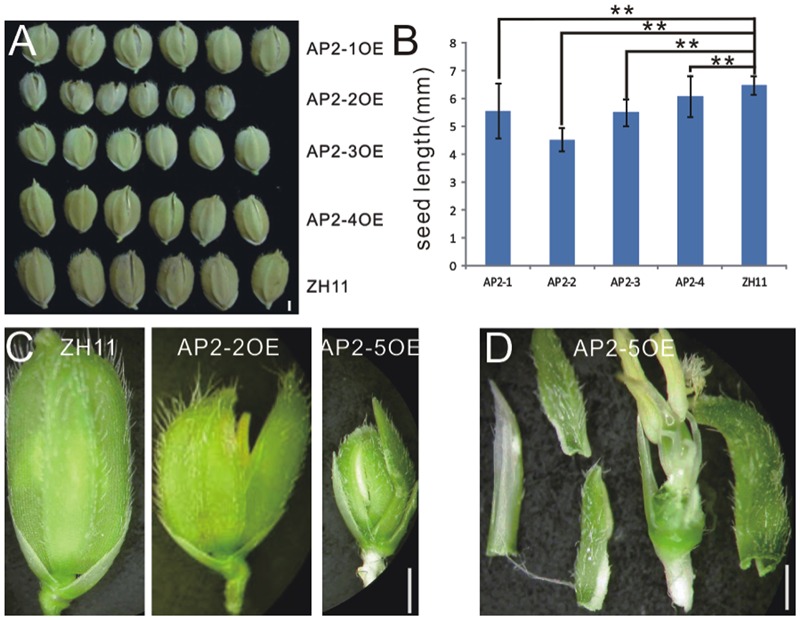
**Phenotypes in the grain and spikelet of *AP2*s overexpressing plants. (A)** Grains of the AP2OEs plants. Bar was 1 mm. **(B)** The grain length of the AP2OEs. Double asterisks represent significant difference determined by the Student’s *t*-test at ^∗∗^*P* < 0.01, respectively. **(C)** Spikelet of ZH11, AP2-2OE, and AP2-5OE plants. **(D)** Dissection of a spikelet of the AP2-5OE plant. Bars in **(C,D)** were 1 mm.

In contrast to miR172aOEs plants, all AP2OE plants showed shortened lemma and palea, and so that reduced grain size (**Figure 3A**), except that AP2-5OE plants were infertile (**Figures 3C,D**). As a result, the 1000-grain-weight of AP2-1OE, AP2-2OE, AP2-3OE, and AP2-4OE decreased (Supplementary Figure S5A), with the correspondingly reduced starch granules (Supplementary Figures S5B–F). AP2-2OE and AP2-5OE showed the most severe abnormality in floral organs. The lemma/palea was shortened appropriately one third in AP2-2OE and more in AP2-5OE (**Figures 3B,C**). Furthermore, in the AP2-2OE, the lemma and palea developed inconsistently, with the palea longer than the lemma (**Figure 3C**), contrasting to those in the miR172OEs (**Figure 1A**); in the AP2-5OE plants, the lemma/palea was malformed (**Figure 3D**). So that miR172/AP2 regulated floral organ identity and flower determinacy, especially elongation of the lemma/palea.

**FIGURE 4:**
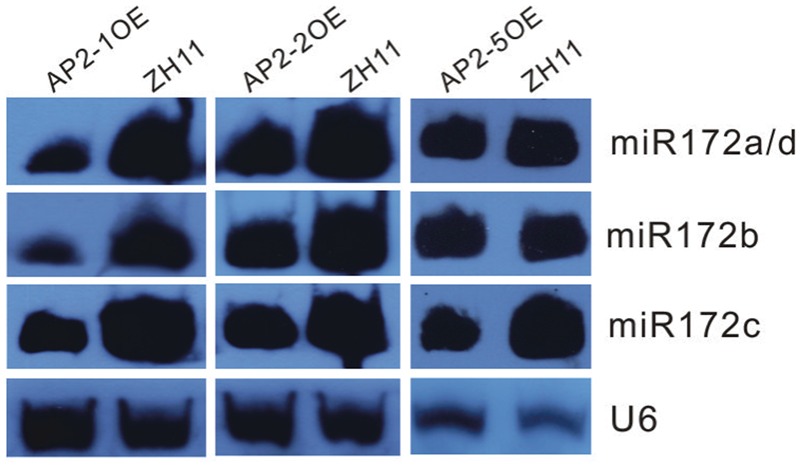
**Northern blot analysis of miR172s in several AP2OE lines.** Expression of respective miR172s was examined in the young panicles of the AP2-1OE, AP2-2OE, and AP2-5OE plants, respectively.

Furthermore, miR172s were down-regulated in the AP2-1OE, AP2-2OE, and AP2-5OE plants (**Figure 4**), indicating the possibility of negative feedback regulation between these *AP2*s and miR172.

### Expression of miR172s Was Inhibited by the *OsMADS1* Gene

The *OsMADS1* gene regulates floral organ identity, affecting lemma/palea development and spikelet determinacy (Jeon et al., 2000; Prasad et al., 2005; Wang K. et al., 2010). The phenotypic similarity of miR172OEs and the *osmads1* mutant suggests a potential interaction between *OsMADS1* and miR172.

**FIGURE 5:**
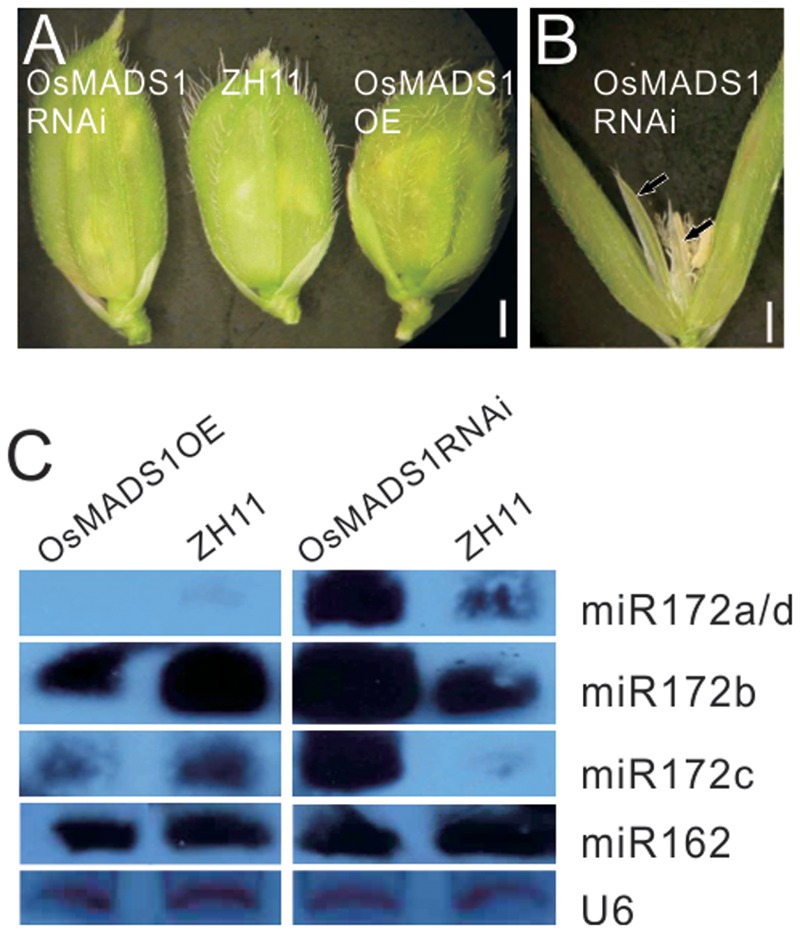
**Analysis of OsMADS1OE and OsMADS1RNAi lines. (A)** Phenotype of OsMADS1RNAi, ZH11, and Osmads1OE plants. **(B)** A floret of OsMADS1RNAi plants. **(C)** Expression of miR172s in the Osmads1OE and OsMADS1RNAi plants.

To obviate the influence of the genetic background on the phenotype, we further constructed *OsMADS1* over expression (OsMADS1OE) and RNAi transgenic plants (OsMADS1RNAi) in the ZH11 background; for each transgenic events, over 90% transformation efficiency were got. In the OsMADS1RNAi plants, the paleas and lemmas were slightly longer than those of the wild-type, and the lemma was longer than the palea (**Figure 5A**). Similarly, OsMADS1RNAi showed open hull and some ectopic glumes developed between whorl1 and whorl2 (**Figure 5B**, arrowhead). So that OsMADS1RNAi could mimic the *osmads1* mutant, although to a milder degree.

All miR172s were down-regulated in the Osmads1OE plants, but up-regulated in the OsMADS1RNAi plants (**Figure 5C**). Expression of miR162 in these plants was basically the same (**Figure 5C**), indicating that the expression of miR172s was specifically modulated by *OsMADS1*.

MADS-box genes regulate flower organ identity by binding to the *cis*-regulatory elements in the target genes termed “CArG-boxes” [consensus 5′CC(A/T)_6_GG3′] (Riechmann et al., 1996; Cui et al., 2010). In the 3 Kb promoter region of miR172a, miR172b, miR172c, and miR172d, there are 6, 4, 3, and 3 CArG-boxes, respectively (Supplementary Figure S6A). We carried out yeast one-hybrid and did not detect the direct binding of OsMADS1 protein to the motifs in the promoters of miR172 (Supplementary Figure S6B). However, OsMADS1 could interact with AP2-2, AP2-3, and AP2-5 in yeast two-hybrid systems (Supplementary Figure S7).

### Expression Character of miR172 and *OsMADS1* during Floral Organ Development.

**FIGURE 6:**
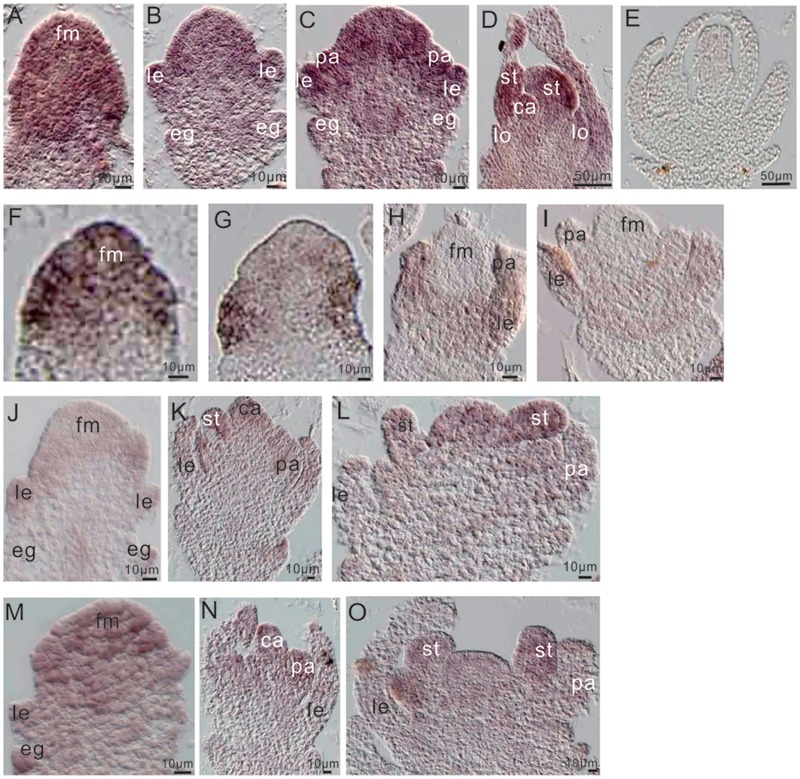
**Expression of miR172 and *OsMADS1* in the process of flower development. (A–D)** and **(F–I)** Respective miR172a and *OsMADS1* expression during floral organ development. **(E)** Sense probe. **(J–L)** and **(M–O)** miR172a expression in Osmads1OE and OsMADS1RNAi plants, respectively, during floral organ development. Abbreviations: fm, floret meristem; pa, palea; le, lemma; lo, lodicules; st, stamen; ca, carpel.

To further examine the relationship between miR172a and *OsMADS1*, we performed *in situ* hybridization of them in flower development. Before differentiation of the floral organs, both *OsMADS1* and miR172a expressed highly at the floret meristem (**Figures 6A,F**); thereafter, miR172a gradually accumulated on all the floral organs, with higher expression in the inner stamens and carpels, and lower expression in the outer palea and lemma (**Figures 6B–E**). However, *OsMADS1* mRNA gradually concentrated on the palea and lemma (**Figures 6G–I**). So that the expression region of *OsMADS1* and miR172 showed complementary character, *OsMADS1* might repress excessive accumulation of miR172 in the lemma and the palea.

Assessment of the expression of miR172a in the Osmads1OE and OsMADS1RNAi lines showed that during development of the empty glume and palea/lemma, miR172a was down-regulated in the OsMADS1OE lines (**Figure 6J** as compared with **Figure 6M**); during the development of inner floral organs, miR172a expressed similarly in the OsMADS1OE and OsMADS1RNAi lines (**Figure 6K** as compared with **Figure 6N**, **Figure 6L** as compared with **Figure 6O**). Therefore, during the developmental process of palea/lemma, *OsMADS1* exhibited inhibition to miR172, which may further explain the longer palea/lemma of miR172OEs plants.

### Overexpression of AP2-2 and AP2-3 Rescued the Elongated Palea/Lemma in the OsMADS1RNAi Plants

Since the miR172aOE, miR172cOE, and miR172dOE were totally infertile, and miR172bOE barely produced any seeds, these lines were not amenable to genetic manipulation, we used *AP2* genes to perform genetic complementation.

**FIGURE 7:**
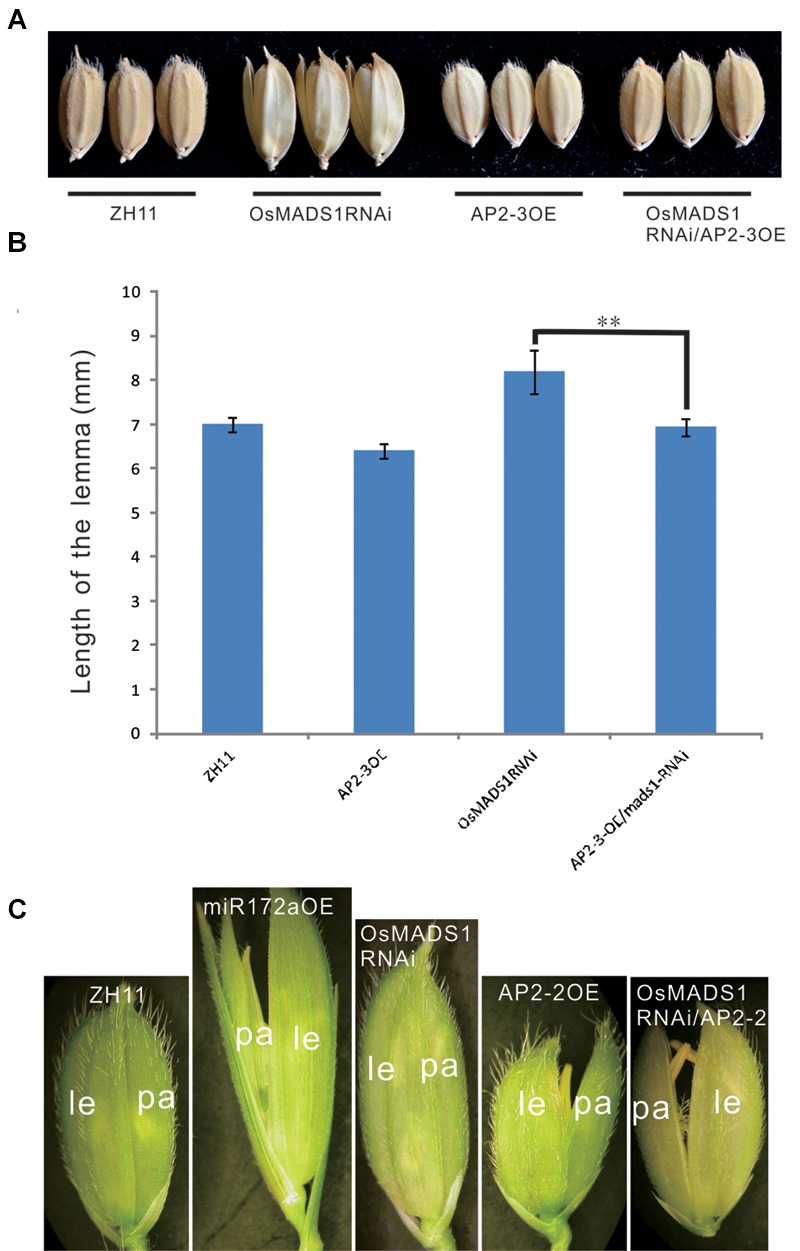
**Phenotypic rescue of OsMADS1RNAi plants by AP2-3OE and AP2-2OE. (A)** Phenotype of the grains of the AP2-3OE/OsMADS1RNAi hybrids. **(B)** The grain length of the OsMADS1RNAi, AP2-3OE/OsMADS1RNAi, and the wild-type. Double asterisks represent significant difference determined by the Student’s *t*-test at ^∗∗^*P* < 0.01, respectively. **(C)** Florets of AP2-2OE transformed into the OsMADS1RNAi plants. Pictures were arranged according to the relative size of the florets. Abbreviations: le, lemma; pa, palea.

First, we made a cross between AP2-3OE and OsMADS1RNAi plants. Among the 11 hybrids of AP2-3OE/OsMADS1RNAi in the F1 generation, six individuals showed shortened and closed hull relatively to the OsMADS1RNAi plants (**Figures 7A,B**), the elongated and inconsistent palea/lemma in OsMADS1RNAi plants could be rescued by *AP2-3* over expression (**Figures 7A,B**).

Next, the AP2-2OE plasmid was genetically transformed into the OsMADS1RNAi plants. The lemma was longer than the palea in both the miR172aOE and OsMADS1RNAi plants, but shorter than the palea in the AP2-2OE plants. In the AP2-2/OsMADS1RNAi plants, the palea/lemma developed synchronously, and they were shorter than those of the OsMADS1RNAi plants but similar to those of the wild-type (**Figure 7C**). These results indicated the elongated and inconsistent palea/lemma in the OsMADS1RNAi plants could be rescued by *AP2-2* over expression.

## Discussion

miR172 was first reported to regulate floral organ development by negatively regulating the *AP2* gene at the post-translation level in *Arabidopsis* (Chen, 2004), and miR172 could cooperate with miR156 to regulate flowering time sequentially (Wu et al., 2009). Various studies showed that miR172 is a pivotal regulator of reproductive development in plants. Here, we studied the function of miR172 in regulating rice floral organ development. In rice, miR172 expressed in each whorl of the floral organs (**Figures 6B,C**), suggesting its function in all these whorls, as manifested by the phenotype of miR172s over expression (**Figure 1**). In miR172aOEs plants, both the palea and the lemma were malformed and elongated, with the lemma longer than the palea, while in the AP2-2OE lines, the lemma was shorter than the lemma, overexpression of all target *AP2* genes resulted in shortened lemma/palea, indicating miR172/AP2s module regulated the elongation and synchronous development of the palea and the lemma in rice. In some cases, miR172aOEs resulted in a “flower-in-flower” phenotype (**Figure 1I**) and a totally inverted process of flower development (**Figure 1J**), indicating an indeterminacy state in flower development. Correspondingly, mutation in the targets of miR172, such as the *SNB* gene and the *OsIDS1* gene resulted in loss of flower determinacy (Lee et al., 2007; Lee and An, 2012).

In *Arabidopsis*, miR172 regulates the elongation of the valve under the modulation of the *FUL* gene (a MADS-box gene) and the *ARF6/8* gene (José Ripoll et al., 2015). Here we showed that miR172 regulated floral organ development in rice, especially the elongation of the palea and lemma, under the modulation of *OsMADS1*. Therefore, the upstream regulatory pathway of miR172 in *Arabidopsis* and rice showed some similarity.

Previous studies revealed that plant miRNAs can act either upstream or downstream of transcription factors. For example, in *Arabidopsis* the transcription factors SQUAMOSA BINDING PROTEIN-like 9 (SPL9) and the MADS-box SHORT VEGETATIVE PHASE (SVP) act as a direct activator and a direct repressor, respectively, of miR172 (Wu et al., 2009; Cho et al., 2012). *OsMADS1* expressed mainly in the palea/lemma and lodicules (**Figure 6H**), while miR172 expressed highly in the newly formed stamens and carpels (**Figure 6D**). Therefore, the expression domains of *OsMADS1* and miR172 showed some degree of complementation, implying the inhibition of *OsMADS1* to miR172. In OsMADS1RNAi plants, the lemma and palea showed inconsistent development, and some ectopic glumes formed between the palea/lemma and the lodicules (**Figure 5B**), which was similar to the pattern in miR172OEs lines. Furthermore, miR172OEs lines showed “flower-in-flower” structures similar to those in the *osmads1* mutant (Wang K. et al., 2010). These phenotypic resemblances indicated the genetic connection between *OsMADS1* and miR172, and our study indicated the inhibition of *OsMADS1* to miR172. However, we did not find direct binding of OsMADS1 to any of the miR172 promoters by yeast one-hybrid system. OsMADS1 was reported to form homo-dimer and hetero-dimer with OsMADS7 and OsMADS8 (Cui et al., 2010). Different OsMADS proteins might form functional complex. Therefore, the binding of OsMADS1 to the promoter of miR172 might require the involvement of several other OsMADS proteins, making it more difficult to be detected. Another possibility is that, OsMADS1 might interact with AP2, and AP2 regulate the expression of miR172 at the transcription level, just as that in *Arabidopsis*, the miR172 repression orchestrated by LUG and SEU co-repressors is dependent on the miR172 target gene *AP2* itself, by a positive-feedback loop allowing *AP2* to maintain its own expression in the outer floral whorls (Grigorova et al., 2011), or by binding to the miR172b promoter (Yant et al., 2010).

**FIGURE 8:**
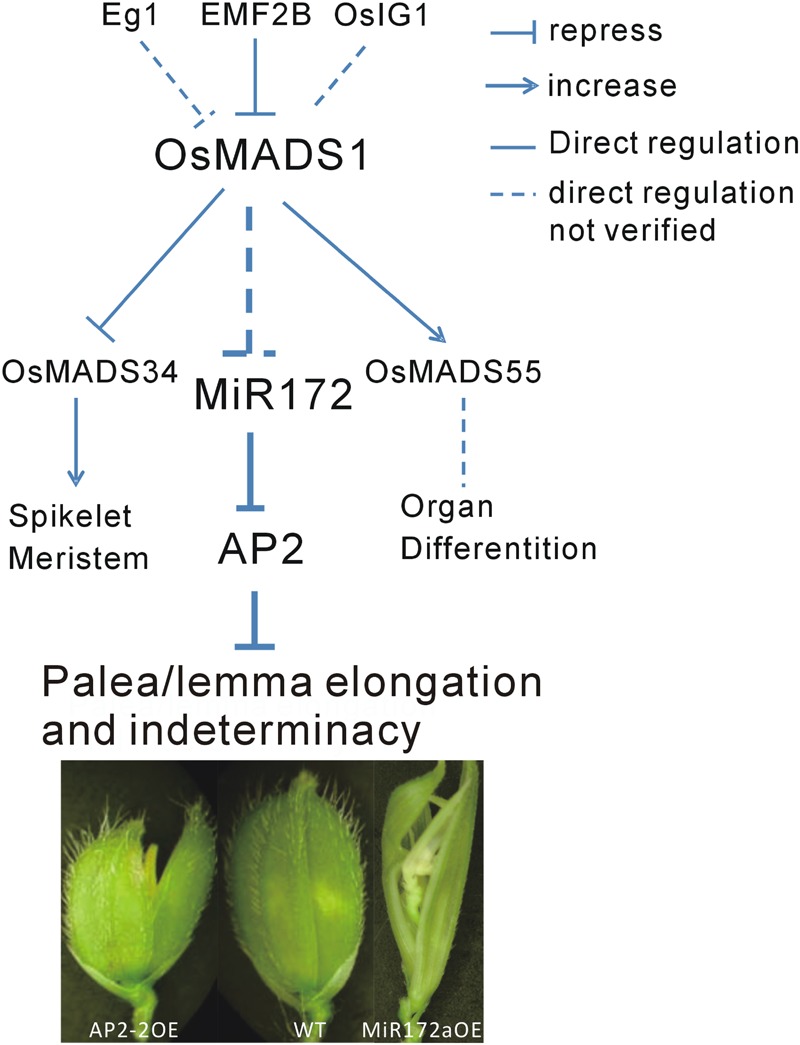
**Diagram of the possible pathway of OsMADS1-miR172-AP2 regulation on palea/lemma.** *OsMADS1* might negatively regulated miR172s, when *OsMADS1* was down-regulated or mutated, miR172s were up-regulated, and the flower showed elongated palea/lemma and indeterminacy. Meanwhile, miR172 negatively regulated its target *AP2* genes, and up-regulation of *AP2* showed shortened palea/lemma (exemplified by AP2-2OE).

Floral organ development is a fundamental event in plant development, especially for plant reproduction. In addition to *OsMADS1*, many genes are involved in this process. Among them, *Extra Glume1* (*EG1*) showed a phenotype with high similarity to that of *osmads1*, and further analysis revealed that *EG1* gene is required for the maintenance of *OsMADS1* expression in the floral meristem and act as an upstream regulator of *OsMADS1* (Li et al., 2009). Also, the polycomb group gene *EMF2B* is a direct repressor of *OsMADS1* (Conrad et al., 2014). A LBD-like transcription factor, *OsIG1*, which mainly regulate female gametophyte development, might be another upstream regulator of *OsMADS1* (Zhang et al., 2015). In the present study, we found that *OsMADS1* functioned by modulating miR172s, indicating one primary pathway consisting of *OsMADS1-miR172-AP2* involved in the regulation of floral organ development (**Figure 8**).

## Author Contributions

Experimental design: XM and ZS; Experiments: ZD, JW, and MZ; Data analysis: JW and ZS; Manuscript preparation: ZS and XM; Supervision, funding and reagents: ZS and XM.

## Conflict of Interest Statement

The authors declare that the research was conducted in the absence of any commercial or financial relationships that could be construed as a potential conflict of interest.

## Footnotes

1 http://rice.plantbiology.msu.edu/

2 https://www.ncbi.nlm.nih.gov/

3 http://sundarlab.ucdavis.edu/smrnas/

4 http://bioinformatics.cau.edu.cn/PMRD/
