# Supplementary material for: OsMADS1 Represses microRNA172 in Elongation of Palea/Lemma Development in Rice
Source: Front Plant Sci. 2016 Dec 20;7:1891. doi: 10.3389/fpls.2016.01891 (PMC5167762; doi:10.3389/fpls.2016.01891)
Supplement: Supplementary file 2 [file Data_Sheet_1.DOCX]

**Title: *OsMADS1* represses microRNA172 in elongation of palea/lemma development in Rice**

Zhengyan Dai, Jiang Wang, Mulan Zhu, Xuexia Miao, Zhenying Shi*

Affiliation for all authors: Key Laboratory of Insect Developmental and Evolutionary Biology, Institute of Plant Physiology and Ecology, Shanghai Institutes for Biological Sciences, Chinese Academy of Sciences, Shanghai, China

*For correspondence:

Zhenying Shi

zyshi@sibs.ac.cn

Phone: +86-21-54924217, Fax: +86-21-54924015

Running title: Comprehensive study of rice miR172.


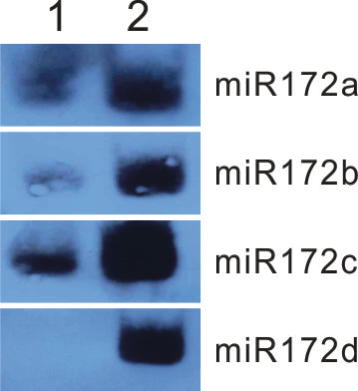


**Figure S1. Verification of the miR172OEs lines by smRNA Northern blotting.**

(1) ZH11. (2) Respective miR172 overexpressing lines. Excatly 20ug total RNA for each sample was loaded for detection.


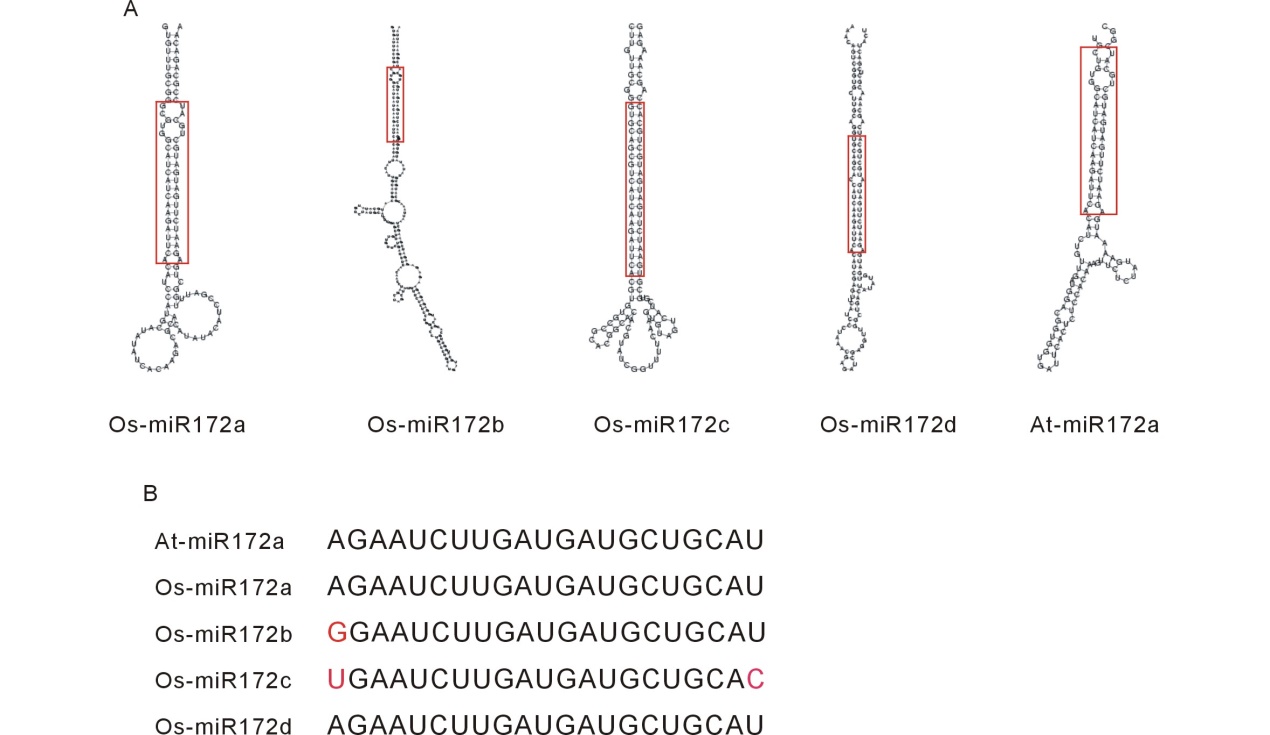


**Figure S2. The miR172s in rice and miR172a in *Arabidopsis.***

(A) The presuors of miR172 in rice and miR172a in *Arabidopsis*. The mature miR172s producing region was indicated by the rectangles. (B) The mature miR172s in rice and miR172a in *Arabidopsis*. The red nuclear tides indicated the different ones from those in OsmiR172a.


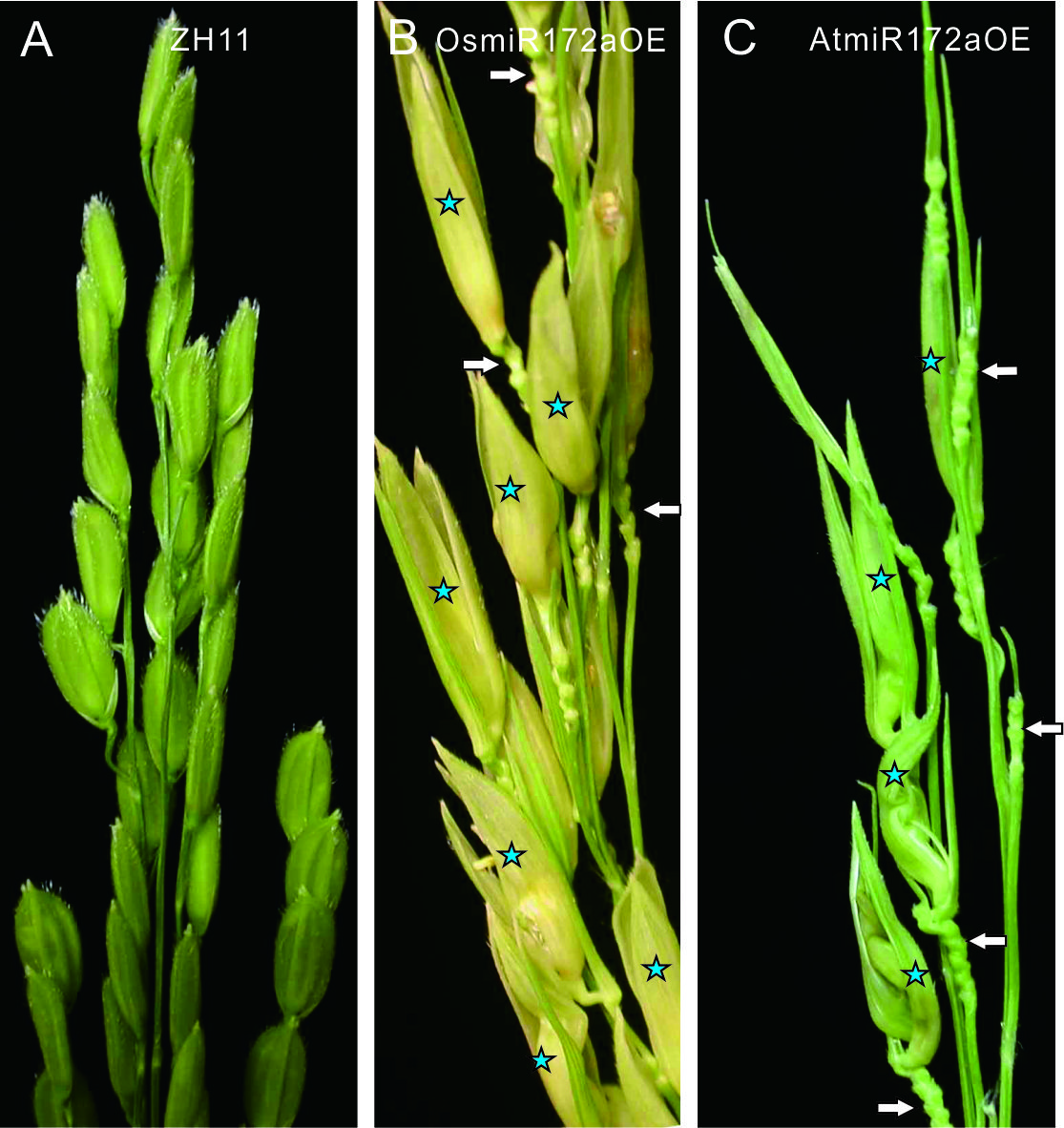


**Figure S3. Conservation of miR172 between different species.**

(A) ZH11. (B) Rice miR172aOE. (C) *Arabidopsis* miR172aOE.


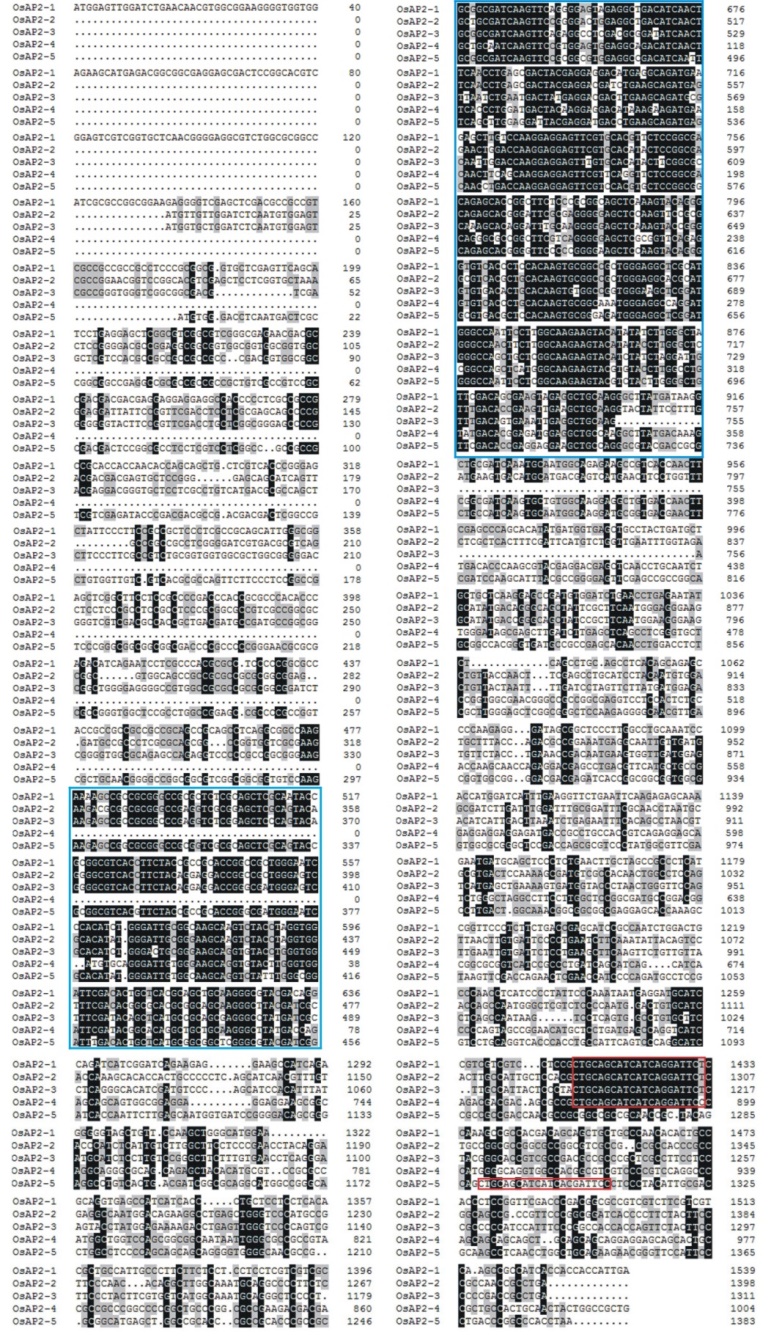


**Figure S4 Alignment of the five miR172 targeting *AP2* genes in rice.**

The two highly conserved AP2 domains were respectively indicated by the blue rectangles and the miR172 binding sites were indicated by red rectangles.


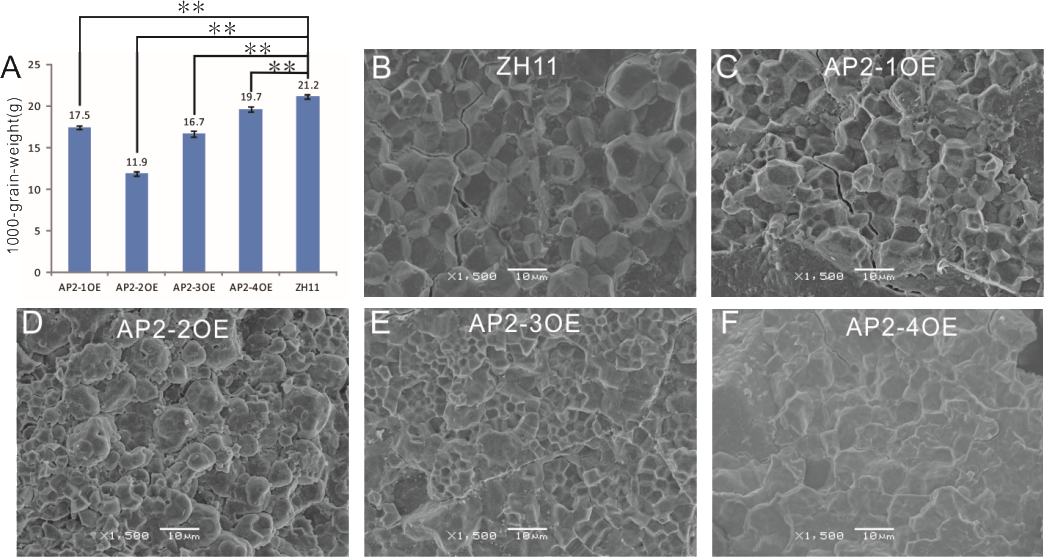


**Figure S5. Grain character of AP2s overexpressing lines.**

(A) 1000-grain-weight of AP2sOE, data was collected from grains without husk. Double asterisks represent significant difference determined by the Student’s *t*-test at ***P*<0.01. (B--F) SEM analysis of the starch granules in ZH11, AP2-1OE, AP2-2OE, AP2-3OE and AP2-4OE.


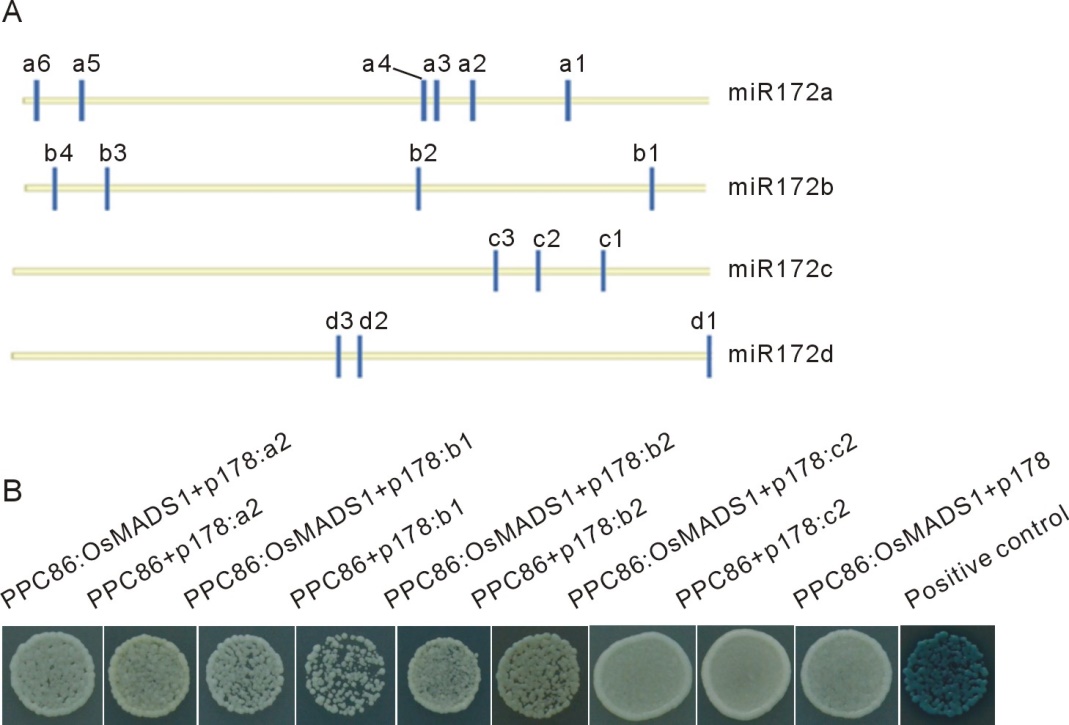


**Figure S6** **Sketch map of the OsMADS1 binding motifs (upright lines) in the promoter (3Kb) of miR172s (A) and yeast one-hybrid of OsMADS1 to these motifs (B).**

A, in the promoter of miR172a, there are 6 sites (a1, a2, a3, a4, a5, a6) for MADS protein binding, and in the promoter of miR172b, there are 4 (b1, b2, b3, b4), and in the promoter of miR172c, there are 3 (c1, c2, c3), and in the promoter of miR172d, there are 3(d1, d2, d3).

B, The OsMADS1 into the pPC86 plasmid, and the genomic fragments containing the MADS1 sites into the p178 plasmid (a2, b1, b2 and c2 as example) respectively, and yeast one hybrid experiment was carried out.


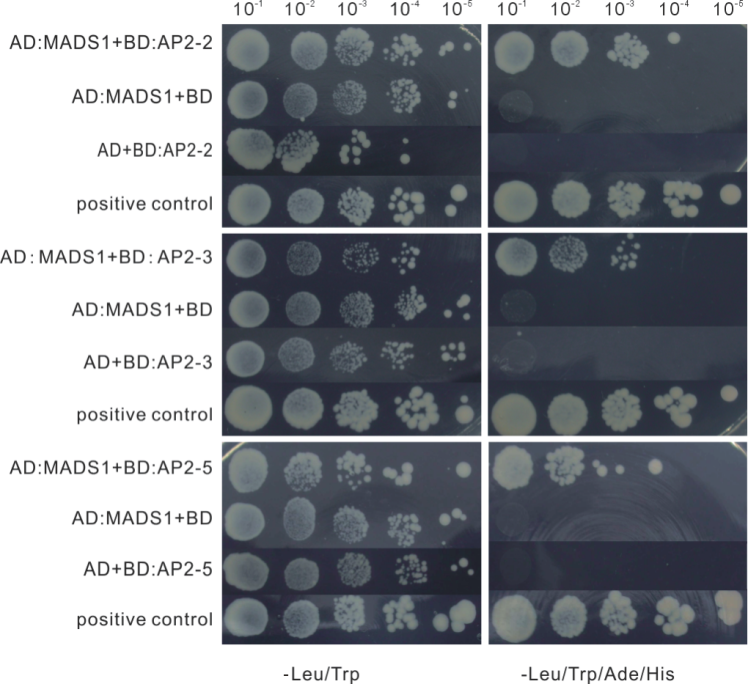


**Figure S7 Yeast two hybrid of OsMADS1 with AP2-2, AP2-3 and AP2-5 respectively.**

Proteins were cloned into the gateway system vector pGADT7 (abbreviated as AD in the figure) and pGBKT7 (abbreviated as BD in the figure). Yeasts were grown on synthetic dextrose (SD) plates without Leu or Trp (SD-LT) and synthetic dextrose plates without Leu, Trp, adenine, or His as indicated.
